# Comparative treatment patterns, healthcare resource utilization and costs of atomoxetine and long-acting methylphenidate among children and adolescents with attention-deficit/hyperactivity disorder in Germany

**DOI:** 10.1007/s10198-016-0836-8

**Published:** 2016-11-05

**Authors:** Peter Greven, Vanja Sikirica, Yaozhu J. Chen, Tammy G. Curtice, Charles Makin

**Affiliations:** 1 Institute of Child and Adolescent Psychiatry, Psychotherapy and Social Pediatrics, Berlin, Germany; 2 H:G University of Health and Sport, Technology and Arts, Berlin, Germany; 3 Shire, Wayne, PA USA; 4 IMS Health, Inc, Fairfax, VA USA

**Keywords:** Atomoxetine, Methylphenidate, Attention-deficit/hyperactivity disorder, Cost of illness, I1 Health

## Abstract

**Background:**

Attention-deficit/hyperactivity disorder (ADHD) imposes a substantial burden on patients and their families.

**Objective:**

A retrospective, propensity score-matched cohort study compared treatment patterns, healthcare resource utilization (HRU) and costs among children/adolescents with ADHD aged 6–17 years at treatment initiation (index) in Germany who received atomoxetine (ATX) or long-acting methylphenidate (LA-MPH) monotherapy.

**Methods:**

Patients received at least one prescription for their index medication (ATX/LA-MPH) during 2006–2010; the first prescription marked the index date. ATX- and LA-MPH-indexed cohorts were matched 1:1 (*n* = 737); a patient subset was identified that had not received ADHD-indicated medications in 12 months prior to index (novel initiators: ATX, *n* = 486; LA-MPH, *n* = 488). Treatment patterns were evaluated among novel initiators, and HRU and costs among the matched cohorts in the 12 months after index.

**Results:**

No significant differences in baseline characteristics were found between the novel initiator patient subsets. ATX-indexed novel initiators had significantly longer persistence to index medication [mean (standard deviation; SD) days: 222.0 (133.9) vs 203.2 (135.0), *P* = 0.029) but higher switching rates (8.8 vs 5.5 %, *P* = 0.045) than LA-MPH-indexed novel initiators. The total ATX-indexed cohort required more prescriptions [any medication; mean (SD): 20.9 (11.5) vs 15.7 (9.0), *P* < 0.001] and outpatient visits [mean (SD): 10.1 (6.3) vs 8.3 (5.3), *P* < 0.001], and incurred significantly higher total median healthcare costs (€1144 vs €541, *P* < 0.001) versus matched LA-MPH patients.

**Conclusions:**

These real-world data indicate that, among children/adolescents with ADHD in Germany, ATX-indexed patients may require more prescriptions and physician visits, and incur higher total healthcare costs, than matched LA-MPH patients.

## Introduction

Attention-deficit/hyperactivity disorder (ADHD) is a common childhood neurobehavioural disorder with core symptoms of inattention, hyperactivity and impulsivity [1]. ADHD often persists into adolescence and adulthood, and negatively influences academic, behavioural, emotional and social functioning [1, 2]. Psychiatric comorbidities are frequently present [3].

The management of ADHD in Germany involves non-pharmacological interventions such as behavioural therapy followed by pharmacotherapy [4]. The use of pharmacotherapy versus no treatment or behavioural therapy in children and adolescents with ADHD is cost-effective from a societal perspective [5]. Approximately one-half of the children/adolescents diagnosed with ADHD in Germany receive pharmacotherapy [6].

Methylphenidate (MPH) is the pharmacotherapy most frequently used for ADHD in Europe, and specifically in Germany [6–8]. Various formulations of long-acting (LA)-MPH and short-acting (SA)-MPH are available [6, 9]. SA-MPH requires multiple daily dosing and has a number of potential limitations, including uneven coverage through the day, and stigmatization by peers as administration of medication is required at school [9]. LA-MPH formulations, with a maximum duration of effect of 8–12 h, are administered once daily and, consequently, may avoid some of the limitations of SA-MPH [9, 10].

Atomoxetine (ATX; Strattera, Lilly, Indiana, IN) is a non-stimulant pharmacotherapy recommended for the treatment of ADHD that has been available in Germany since March 2005 [6]. It may be used for the treatment of patients who respond suboptimally or are intolerant to stimulant therapy, or have comorbidities such as tic disorders [1, 11]. ATX may also be used when there are concerns about substance misuse, or if patients express a specific treatment preference [4, 11].

ADHD imposes a substantial economic burden on individuals, their families, and healthcare systems [12–16]. A total direct annual cost of €3888 per patient (mean age 15 years) was reported using claims data for 2008 from a major German health insurance fund [12]. This represents an additional cost of €2902 versus age- and sex-adjusted controls without ADHD [12]. Moreover, healthcare costs associated with ADHD are increasing [6, 12, 16, 17]. As estimates of ADHD prevalence have not increased over time [18], the increasing costs are likely due to improved identification of affected patients and more intensive management [16]. However, data on comparative treatment patterns and associated economic burden among patients with ADHD who receive ATX and LA-MPH in Germany are sparse [6].

This study was designed to specifically compare pharmacotherapy treatment patterns, healthcare resource utilization (HRU; including pharmacy prescriptions, outpatient visits and inpatient admissions), and associated costs among children and adolescents with ADHD who received ATX or LA-MPH monotherapy.

## Methods

### Data

This retrospective cohort study was conducted using an electronic medical records (EMR) database, IMS Disease Analyzer (IMS Health, Fairfax, VA), for Germany. The IMS Disease Analyzer comprises longitudinal patient-level data with more than 15 million anonymized patient records from approximately 3000 office-based physicians in Germany; the database is sampled using summary statistics from all doctors in Germany as published annually by the German Medical Association [19, 20]. The distribution of patients in the database is similar to the overall population distribution and provides a nationally representative and validated sample of all major German geographic regions [19].

EMR data collected from physicians in general/internal medicine (including primary care), paediatrics, psychiatry and neurology between 1 January 2005 and 31 December 2011 were used. Longitudinal, patient-level data were available, including information on demographic characteristics, medical diagnoses (coded using International Classification of Diseases, Tenth Revision) and details of prescribed medications.

### Sample selection

Patients received at least one prescription for ATX (Strattera) or LA-MPH (any formulation) between 1 January 2006 and 31 December 2010 (patient selection window). The date of the first prescription for ATX or LA-MPH monotherapy for a patient during this window was defined as the index date, and the treatment received defined as the index medication.

Patients were 6–17 years of age at index, had available data (including a recorded diagnosis of ADHD) from physician visits during the 12 months prior to (baseline period) and the 12 months after (follow-up period) the index date, and had received at least one prescription for their index medication during the follow-up period; they may have received prescriptions for their index medication in the baseline period.

Patients were excluded from the study if they had received prescriptions for both LA-MPH and ATX at index or within 60 days prior to index (concurrently or sequentially), or had received SA-MPH at or within 60 days prior to index; patients were not excluded for receiving any other concomitant medications.

Patients were categorized into one of two mutually exclusive treatment groups based on their index medication. Eligible patients who received both ATX and LA-MPH monotherapy during the selection window were preferentially included in the ATX-indexed group to maximize the ATX sample size. In order to mitigate biases from ongoing treatment users, a subset of novel initiators (i.e. without treatment in the baseline period) was used to evaluate treatment outcomes.

#### Matched treatment cohorts

Propensity score matching was used to account for observed differences between treatment cohorts. Patients in the ATX-indexed cohort were matched 1:1 (with a calliper of 0.0001) to patients in the LA-MPH-indexed cohort using a ‘nearest neighbour’ greedy matching algorithm [21].

The dependent variable in the propensity score model was the likelihood of receipt of a prescription for ATX. The following covariates were included: age (6–12 years or 13–17 years), sex, index year, geographical region, and physician practice specialty at baseline; and comorbidities, medication use, ADHD-indicated medication-naïvety, and number of outpatient visits and inpatient admissions during the 12-month baseline period. The goodness of fit of the model was evaluated using the Hosmer–Lemeshow test and analysis of residuals [22].

The quality of the match was assessed by graphically comparing the overlap between the estimated propensity score of matched and unmatched patients [22]. Generalized linear models with negative binomial (for HRU) and gamma (for healthcare costs) distributions were used to verify if any residual differences in baseline variables remained between the matched treatment cohorts.

#### Novel initiator subset

We identified a subset of patients from each of the matched treatment cohorts who had not received any ADHD-indicated medications (ATX, LA-MPH or SA-MPH) during the 12-month baseline period. These patients were termed ‘novel initiators’; data were used to assess treatment patterns.

### Outcome measures

Demographic and clinical characteristics recorded during the baseline period or at index were retrieved. Treatment patterns, HRU, and associated healthcare costs were evaluated.

#### Treatment patterns

Treatment patterns during the 12-month follow-up were compared only in the ATX- and LA-MPH-indexed novel initiator subsets in order to minimize the effect of ongoing treatment. Outcomes included treatment persistence on index medication, discontinuation, switching, restarting and augmentation, and are defined in Table 1. Switching to and/or augmentation with medications for psychiatric disorders (antipsychotics, tranquilizers, antidepressants and mood stabilizers, psycholeptic–psychoanaleptic combinations, anticonvulsants, hypnotics/sedatives and clonidine) were also evaluated.

**Table 1:** Treatment outcomes

Outcome	Definition
Persistence	The number of continuous days of medication from index until discontinuation, switching, augmentation or the end of the follow-up period, whichever occurred first
Discontinuation	A gap in index therapy of at least 30 days following the last day of supply of the previous prescription. The date immediately following the 30-day gap was considered to be the discontinuation date. However, a gap of up to 90 days was permitted in May, June and July to allow temporary suspension of medication during so-called ‘drug holidays’ [23]
Switching^a^	Initiation of an ADHD- or other mental health-indicated medication within 30 days after discontinuation of the index medication. A supply of 30 days or more of the new non-index ADHD medication was required. Only the first switch was evaluated
Restarting^a^	Provision of a new prescription for the index medication after the switch/discontinuation date but before the end of the follow-up period
Augmentation^a^	Addition of a non-index medication, with at least 30 days of concurrent use with the index medication. Only the first augmentation within the 12-month follow-up period was evaluated

* ADHD* attention-deficit/hyperactivity disorder

^a^Assessed only among patients who discontinued or switched their index medication

#### HRU and total healthcare costs

Per-person HRU and the associated costs incurred by patients with ADHD who received pharmacotherapy during the 12-month follow-up were compared in the overall matched ATX- and LA-MPH-indexed cohorts.

The following measures were used to evaluate HRU: pharmacy prescriptions including ADHD-indicated medications, medications for other mental health disorders and other (excluding ADHD and mental health-related) medications; outpatient visits to physicians in general/internal medicine (including primary care), paediatrics, psychiatry and neurology; inpatient admissions; and sick notes (to excuse patients from work or school).

As the EMR database does not include cost information, the direct medical costs were calculated from a public reimbursement perspective by applying standardized unit costs (for prescriptions, outpatient visits and inpatient admissions) to quantities of per-person HRU. Unit costs were based on standardized mean reimbursement rates from a series of official German tariffs. Pharmacy prescriptions costs were obtained from the 2011 Rote Liste [24]. The 2011 *Einheitlicher Bewertungsmaßstab* (EBM; Uniform Valuation Scheme) doctors’ fee scale was used to calculate outpatient costs. A uniform orientation value of €0.035 per point (attributed according to the EBM) was assumed to derive unit costs per outpatient visit by type, which is consistent with guidance from the German *Kassenärztliche Bundesvereinigung* (National Association of Statutory Health Insurance Physicians) [25]. Inpatient unit costs were obtained from the 2012 German Diagnosis-related Group catalogue; the weighted average cost was calculated based on the diagnosis and reason for hospitalization [26]. For consistency, all costs were reported in 2012 German Euros using the Harmonised Index of Consumer Prices [27].

### Statistical analysis

Patients were grouped by index therapy and all analyses were performed on an intent-to-treat basis. Descriptive statistics were used to assess differences between patients in the ATX- and LA-MPH-indexed cohorts before propensity score matching the groups. Specifically, Pearson Chi squared tests were used to compare categorical variables and Wilcoxon rank-sum tests were used to compare continuous variables in the pre-match sample.

Outcome data are reported using descriptive statistics. The duration of index medication persistence was evaluated using the log-rank Chi squared test. The Chi squared test (for categorical variables) and *t* test or Wilcoxon–Mann–Whitney test (for continuous variables) were used to assess other differences between the two groups. All statistical tests were two-tailed at an alpha level of *P* = 0.05 and choice of test type was based on data distribution (e.g. normality); no multiplicity adjustment was performed.

## Results

### Study population

**Fig. 1:**
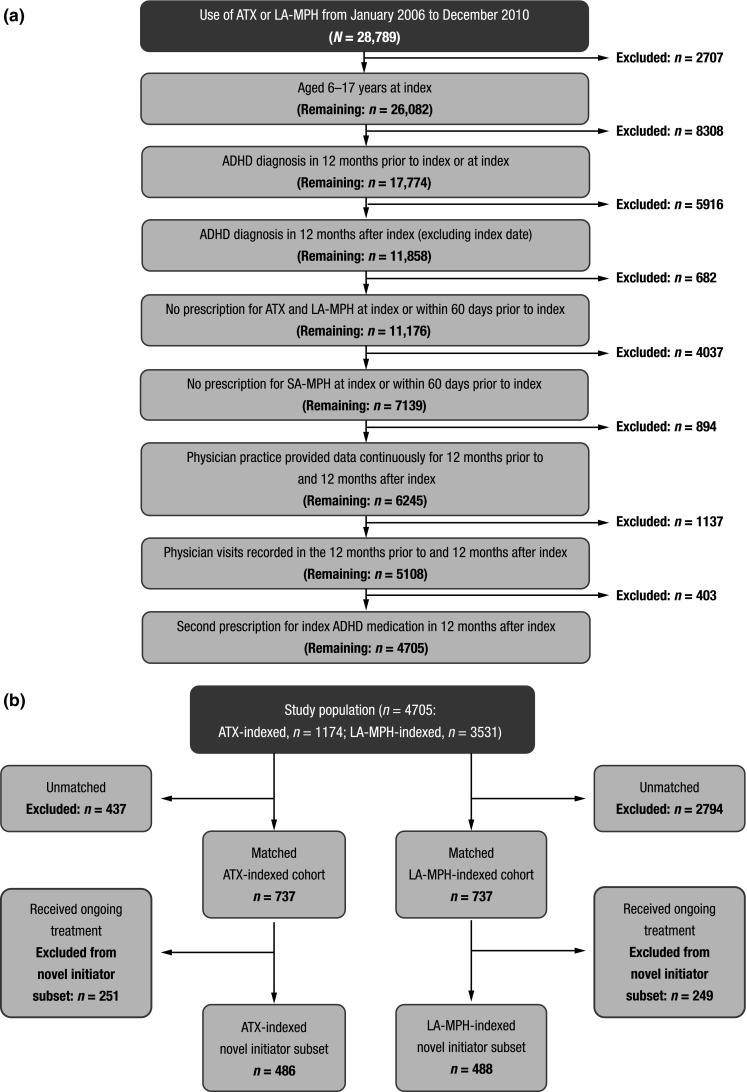
Study population. **a** Patient selection, SA-MPH short-acting methylphenidate. **b** Matched atomoxetine (ATX)- and long-acting methylphenidate (LA-MPH)-indexed patients

A total of 28,789 patients received at least one prescription for ATX or LA-MPH during the patient selection window (Fig. 1a). Of these, 4705 met all inclusion criteria and comprised the study population (Fig. 1b). The ATX-indexed group (*n* = 1174) was approximately threefold smaller than the LA-MPH-indexed group (*n* = 3531).

Baseline demographic characteristics were similar in the two groups; significant differences were observed only in geographic region (*P* < 0.001) and physician practice specialty (*P* < 0.001). Significant differences between the two treatment cohorts were found in a number of clinical characteristics. At index, the ATX-indexed cohort had a higher proportion of patients who were ADHD-indicated medication-naïve (during the 12-month baseline period) than did the LA-MPH-indexed cohort [764/1174 (65.1%) vs 2134/3531 (60.4%), *P* = 0.005], but a lower proportion of patients who were index medication-naïve [1008/1174 (85.9%) vs 3143/3531 (89.0%), *P* = 0.004]. The ATX-indexed cohort also had a higher proportion of patients who were MPH medication-naïve at index [896/1174 (76.3%) vs 2140/3531 (60.6%), *P* < 0.001]. Over the baseline period, the ATX-indexed cohort received a higher mean number of prescriptions for any medication than did the LA-MPH-indexed cohort (8.7 vs 7.4, *P* < 0.001).

#### Matched treatment cohorts

A total of 737 patients from the ATX-indexed cohort were matched with the same number of patients from the LA-MPH-indexed cohort (total *n* = 1474; Fig. 1b). The two treatment cohorts matched within decile subgroups (*P* = 0.652), indicating that the propensity score model was well calibrated.

The baseline demographic and clinical characteristics are presented in Table 2. The majority of patients in the matched cohorts were 6–12 years of age (71.3%) and male (80.3%). Most of the recorded outpatient visits were conducted by paediatricians (61.0%) or psychiatrists (31.6%).

**Table 2:** Baseline demographic and clinical characteristics of the two matched treatment cohorts

Measure	ATX	LA-MPH	Overall	*P* value
	*n* = 737	*n* = 737	*n* = 1474	
Female	153 (20.8)	137 (18.6)	290 (19.7)	0.295
Age group, years				0.527
6–12	531 (72.0)	520 (70.6)	1051 (71.3)	
13–17	206 (28.0)	217 (29.4)	423 (28.7)	
Mean	10.9	11.2	11.1	
SD	2.7	2.6	2.6	
Median	11.0	11.0	11.0	
Region^a^				0.943
I (S–H, HAM, N–S, BRE)	177 (24.0)	166 (22.5)	343 (23.3)	
II (N–W)	239 (32.4)	236 (32.0)	475 (32.2)	
III (HES, SAA, R–P)	98 (13.3)	97 (13.2)	195 (13.2)	
IV (B–W)	29 (3.9)	25 (3.4)	54 (3.7)	
V (BAY)	101 (13.7)	107 (14.5)	208 (14.1)	
VI (B)	23 (3.1)	30 (4.1)	53 (3.6)	
VII (MEC, BRA, S–A)	47 (6.4)	54 (7.3)	101 (6.9)	
VIII (TH, SAC)	23 (3.1)	22 (3.0)	45 (3.1)	
Insurance				0.568
Member	39 (5.3)	39 (5.3)	78 (5.3)	
Dependant (family member)	607 (82.4)	623 (84.5)	1230 (83.4)	
Private	57 (7.7)	44 (6.0)	101 (6.9)	
Retiree	34 (4.6)	31 (4.2)	65 (4.4)	
Practice specialty				0.817
General/internal medicine	48 (6.5)	42 (5.7)	90 (6.1)	
Paediatrics	446 (60.5)	453 (61.5)	899 (61.0)	
Psychiatric	232 (31.5)	234 (31.8)	466 (31.6)	
Neurology	11 (1.5)	8 (1.1)	19 (1.3)	
Comorbid conditions^b^				
Learning difficulties	121 (16.4)	100 (13.6)	221 (15.0)	0.126
Accidental injury	81 (11.0)	88 (11.9)	169 (11.5)	0.567
Anxiety and other neurotic disorders	52 (7.1)	49 (6.6)	101 (6.9)	0.757
Conduct disorder	44 (6.0)	53 (7.2)	97 (6.6)	0.344
Emotional disorders	46 (6.2)	36 (4.9)	82 (5.6)	0.256
Naïve to treatment during the 12-month baseline period				
Naïve to all ADHD-indicated medications—ATX, LA-MPH, SA-MPH	486 (65.9)	488 (66.2)	974 (66.1)	0.912
Naïve to all MPH medications	572 (77.6)	489 (66.4)	1061 (72.0)	<0.001
Naïve to index ADHD-indicated medications	637 (86.4)	655 (88.9)	1292 (87.7)	0.154
Pre-index other mental health medications^c^	46 (6.2)	25 (3.4)	71 (4.8)	0.011

Data are given as *n* (%) unless indicated otherwise

*ATX* atomoxetine, *LA*-*MPH* long-acting methylphenidate, *MPH* methylphenidate, *SA*-*MPH* short-acting methylphenidate, *SD* standard deviation

^a^Regions were defined by Ärztestatistik der Bundesärztekammer (physicians’ statistics of the German Medical Association) [28]: *B* Berlin, *BAY* Bayern, *BRA* Brandenburg, *BRE* Bremen, *B–W* Baden–Württemberg, *HAM* Hamburg, *HES* Hessen, *MEC* Mecklenburg–Vorpommern, *N–S* Niedersachsen, *N–W* Nordrhein–Westfalen, *R–P* Rheinland-Pfalz, *S–A* Sachsen–Anhalt, *SAA* Saarland, *SAC* Sachsen, *S–H* Schleswig–Holstein, *TH* Thüringen

^b^Five most commonly reported comorbidities are presented

^c^Pre-index medications among patients with at least one event

No significant differences between the ATX- and the LA-MPH-indexed cohorts were found in: sex, age or the proportion of patients at index who were index medication-naïve or any ADHD-indicated medication-naive (Table 2). Furthermore, no significant differences were found in the prevalence of psychiatric comorbidities or the number of prescriptions (for any medication) received over the baseline period. However, significant differences between the matched cohorts remained in two baseline characteristics: a greater proportion of ATX- than LA-MPH-indexed patients were naïve to all MPH medications (77.6 vs 66.4%, *P* < 0.001) and a greater proportion of ATX-indexed patients received other (non-ADHD) mental health medications during the baseline period (6.2 vs 3.4%, *P* = 0.011).

Multivariate regression analysis was used to adjust for residual differences, by incorporating significant variables [‘Naïve to all MPH medications’ and ‘Received other (non-ADHD) mental health medications during the baseline period’] into regression models to estimate mean values for outpatient visits and total costs as both main effects and interaction terms. The predicted means were similar to the original estimates.

#### Novel initiator subsets

A subset of patients who had not received any ADHD medication during the baseline period was identified from each matched cohort (Fig. 1b). These ‘novel initiator’ subsets comprised 486 ATX-indexed and 488 LA-MPH-indexed patients.

The baseline demographic and clinical characteristics of the two novel initiator subsets are presented in Table 3. Similar to the matched treatment cohorts, the majority of patients in the novel initiator subsets were 6–12 years of age (72.8%) and male (79.6%). No significant differences in baseline characteristics were found between the ATX- and the LA-MPH-indexed novel initiator subsets.

**Table 3:** Baseline demographic and clinical characteristics of the two novel initiator subsets

Measure	ATX	LA-MPH	Overall	*P* value
	*n* = 486	*n* = 488	*n* = 974	
Female	106 (21.8)	93 (19.1)	199 (20.4)	0.287
Age group, years				0.078
6–12	366 (75.3)	343 (70.3)	709 (72.8)	
13–17	120 (24.7)	145 (29.7)	265 (27.2)	
Mean	10.7	11.1	10.9	
SD	2.8	2.6	2.7	
Median	10.0	11.0	11.0	
Region^a^				0.978
I (S–H, HAM, N–S, BRE)	130 (26.7)	119 (24.4)	249 (25.6)	
II (N–W)	161 (33.1)	159 (32.6)	320 (32.9)	
III (HES, SAA, R–P)	46 (9.5)	52 (10.7)	98 (10.1)	
IV (B–W)	21 (4.3)	20 (4.1)	41 (4.2)	
V (BAY)	59 (12.1)	59 (12.1)	118 (12.1)	
VI (B)	23 (4.7)	26 (5.3)	49 (5.0)	
VII (MEC, BRA, S–A)	33 (6.8)	37 (7.6)	70 (7.2)	
VIII (TH, SAC)	13 (2.7)	16 (3.3)	29 (3.0)	
Insurance				0.930
Member	24 (4.9)	24 (4.9)	48 (4.9)	
Dependant (family member)	401 (82.5)	406 (83.2)	807 (82.9)	
Private	40 (8.2)	35 (7.2)	75 (7.7)	
Retiree	21 (4.3)	23 (4.7)	44 (4.5)	
Practice specialty				0.992
General/internal medicine	30 (6.2)	30 (6.1)	60 (6.2)	
Paediatrics	298 (61.3)	301 (61.7)	599 (61.5)	
Psychiatric	152 (31.3)	152 (31.1)	304 (31.2)	
Neurology	6 (1.2)	5 (1.0)	11 (1.1)	
Comorbid conditions^b^				
Learning difficulties	82 (16.9)	65 (13.3)	147 (15.1)	0.122
Accidental injury	57 (11.7)	59 (12.1)	116 (11.9)	0.862
Anxiety and other neurotic disorders	42 (8.6)	38 (7.8)	80 (8.2)	0.627
Conduct disorder	33 (6.8)	35 (7.2)	68 (7.0)	0.815
Emotional disorders	32 (6.6)	30 (6.1)	62 (6.4)	0.780
Pre-index other mental health medications^c^	29 (6.0)	19 (3.9)	48 (4.9)	0.135

Data are given as *n* (%) unless indicated otherwise

^a^Regions defined as in Table 2

^b^Five most commonly reported comorbidities are presented

^c^Pre-index medications among patients with at least one event

### Treatment patterns

All treatment pattern outcomes were evaluated in the novel initiator subset only.

ATX-indexed novel initiators demonstrated significantly longer persistence to their index medication than did LA-MPH-indexed novel initiators [mean (standard deviation; SD) number of days 222.0 (133.9) vs 203.2 (135.0), *P* = 0.029]. LA-MPH-indexed novel initiators had significantly higher index medication discontinuation rates compared with ATX-indexed novel initiators [269/486 (55.4%) vs 285/488 (58.4%), *P* < 0.001]. ATX-indexed novel initiators had significantly higher rates of switching (to a non-index ADHD- or other mental health-indicated medication) than did LA-MPH-indexed novel initiators [43/486 (8.8%) vs 27/488 (5.5%), *P* = 0.045; Table 4]. Specifically, 3.3% (16/486) of ATX-indexed novel initiators switched to LA-MPH and 0.2% (1/488) of LA-MPH-indexed novel initiators switched to ATX. A significantly greater proportion of LA-MPH- compared with ATX-indexed novel initiators restarted their index medication after either discontinuation or switching [171/181 (94.5%) vs 87/126 (69.0%), *P* < 0.001; Table 4]. Similar rates of augmentation (with any medication) were observed among ATX- and LA-MPH-indexed novel initiators [83/486 (17.1%) vs 89/488 (18.2%), *P* = 0.635]. ATX-indexed novel initiators were less likely to augment with SA-MPH than were LA-MPH-indexed novel initiators [23/486 (4.7%) vs 64/488 (13.1%), *P* < 0.001), but more likely to augment with antipsychotics [37/486 (7.6%) vs 16/488 (3.3%), *P* = 0.003].

**Table 4:** Treatment patterns among novel initiators

	ATX	LA-MPH	Overall	*P* value for the difference between ATX- and LA-MPH-novel initiators
	*n* = 486	*n* = 488	*n* = 974	
Patients who had ≥1 switch to a non-index ADHD- or other mental health-indicated medication during the 12-month follow-up period, *n* (%)	43 (8.8)	27 (5.5)	70 (7.2)	0.045
Patients who restarted index medication (after discontinuation or switching) during the 12-month follow-up period, *n* (%^a^)	87 (69.0)	171 (94.5)	258 (84.0)	<0.001
Time from index to first switch or augmentation, *n* (%)	*n* = 126	*n* = 116	*n* = 242	
0–90 days	80 (63.5)	75 (64.7)	155 (64.0)	0.384
91–180 days	26 (20.6)	29 (25.0)	55 (22.7)	
181–360 days	20 (15.9)	12 (10.3)	32 (13.2)	

^a^Denominator is the total number of patients who switched or discontinued (ATX, *n* = 126; LA-MPH, *n* = 181; overall, *n* = 307)

The majority of (first) switches/augmentations occurred within the initial 90 days of treatment among both ATX- and LA-MPH-indexed novel initiators (Table 4). The mean (SD) time to first switch/augmentation of index medication was slightly, although not significantly, longer among ATX- than LA-MPH-indexed novel initiators [97.0 (91.7) days vs 84.3 (79.1) days, *P* = 0.253].

### HRU and total healthcare costs

The HRU and cost analyses reported below were evaluated among the two matched cohorts, which included novel initiators and ongoing treatment users (ATX, *n* = 737; LA-MPH, *n* = 737).

Patients in the ATX-indexed cohort received significantly more prescriptions (for any medication) than did the matched LA-MPH-indexed cohort [mean (SD) number, 20.9 (11.5) vs 15.7 (9.0), *P* < 0.001; Table 5]. ATX-indexed patients received significantly more prescriptions for ADHD-indicated medication than did LA-MPH-indexed patients [mean (SD) number, 9.2 (5.1) vs 6.9 (4.0), *P* < 0.001]. Furthermore, ATX-indexed patients received significantly more prescriptions for both index [8.0 (4.4) vs 6.2 (3.6), *P* < 0.001] and other ADHD-indicated medications [SA-MPH; 4.8 (3.7) vs 2.6 (1.8), *P* < 0.001).

**Table 5:** Per-patient healthcare resource use of the two matched treatment cohorts over the 12-month follow-up period

	ATX	LA-MPH	Overall	*P* value for the difference between matched ATX and LA-MPH cohorts
	*n* = 737	*n* = 737	*n* = 1474	
Mean (SD) number of prescriptions per patient				
All medications	20.9 (11.5)	15.7 (9.0)	18.3 (10.7)	<0.001
Index ADHD medications (ATX, LA-MPH)	8.0 (4.4)	6.2 (3.6)	7.1 (4.1)	<0.001
Other ADHD-indicated medication (SA-MPH)	4.8 (3.7)	2.6 (1.8)	3.7 (3.1)	<0.001
Mean (SD) number of visits per patient				
All outpatient visits	10.1 (6.3)	8.3 (5.3)	9.2 (5.9)	<0.001
Paediatric visits	10.3 (6.1)	8.7 (4.9)	9.5 (5.6)	<0.001
Psychiatric visits	7.0 (4.3)	5.1 (3.3)	6.1 (3.9)	<0.001
General/internal medicine visits	7.7 (5.6)	6.6 (4.8)	7.2 (5.2)	0.279
Neurology visits	2.3 (2.2)	2.2 (1.8)	2.2 (2.0)	0.742
Median ADHD-related healthcare costs, euros				
Total cost	1144	541	779	<0.001
Prescription cost	978	397	618	<0.001
Outpatient cost	124	124	124	0.059
Inpatient cost	0	0	0	NA

Patients in the ATX-indexed cohort required significantly more outpatient visits than did LA-MPH-indexed patients [mean (SD) number, 10.1 (6.3) vs 8.3 (5.3), *P* < 0.001; Table 5]. The mean (SD) number of inpatient admissions was similar in the ATX- and LA-MPH-indexed cohorts [1.13 (0.3) and 1.12 (0.4), respectively; *P* = 0.933]. Sick notes were issued for 0.8% (6/737) of visits in both treatment cohorts.

Total per-person median healthcare costs were significantly higher in the ATX-indexed cohort than the matched LA-MPH-indexed cohort (€1144 ATX vs €541 LA-MPH, *P* < 0.001; Table 5). The greater costs were predominantly because of higher median retail pharmacy expenditures (€978 ATX vs €397 LA-MPH, *P* < 0.001), which were closely related to the cost of index medications (€862 ATX vs €353 LA-MPH, *P* < 0.001).

## Discussion

This retrospective, propensity score-matched cohort study of children and adolescents with ADHD in Germany showed that ATX-indexed novel initiators had longer persistence to their index medication, but were more likely to switch medications compared with LA-MPH novel initiators. The overall ATX-indexed cohort (including novel initiators and ongoing treatment users) required more prescriptions and outpatient visits than did the matched LA-MPH cohort. Accordingly, for the costs analysed, the ATX-indexed cohort incurred higher healthcare costs compared with the matched LA-MPH cohort. The observed cost difference was largely driven by the larger per-patient pharmacy costs of ATX.

LA-MPH-indexed novel initiators were more likely to discontinue treatment than were ATX-indexed novel initiators, but were also more likely to restart their index medication. As it may take up to 12 weeks for the optimal effects of ATX to be achieved [29], patients may choose not to take pharmacotherapy breaks. In contrast, the effects of LA-MPH wane daily, so patients may perceive that they can manage at least temporarily without treatment and then restart LA-MPH when they decide or at the request of a family member/carer. Although it is not specifically advised by German ADHD practice guidelines [4], some clinicians sanction the requests of patients or their parents for frequent LA-MPH treatment breaks over the weekend or during short school holidays.

A greater proportion of ATX-indexed novel initiators switched to LA-MPH compared with LA-MPH-indexed novel initiators who switched to ATX (3.3% vs 0.2%). This is to be expected because, although there are many reasons for starting ADHD treatment with a non-stimulant medication, patients may subsequently switch to MPH as it is more efficacious than ATX [1, 4, 11, 30].

Similar overall rates of treatment augmentation were observed among ATX- and LA-MPH-indexed novel initiators but the medications used for augmentation varied. Concomitant SA-MPH was prescribed more frequently to LA-MPH- than ATX-indexed novel initiators. This may be expected as SA-MPH is sometimes used in clinical practice to supplement the duration of effect of different formulations of LA-MPH [9]. Augmentation with antipsychotic medications was observed more frequently with ATX- than LA-MPH-indexed novel initiators. As in any retrospective research, it is possible that unobserved variables could drive differences in outcomes. For example, if ATX-indexed novel initiators had more severe ADHD or comorbid symptoms that were not recorded (such as aggression) than did LA-MPH-indexed novel initiators, this could be a potential rationale why they received antipsychotic medications. The presence of such comorbidities could ultimately result in higher HRU and costs. Future research should explore whether such relationships occur.

Our findings are broadly in line with previous reports of poor adherence and persistence to ADHD medications, and relatively high augmentation rates [31–34]. However, to our knowledge, this is the first large, population-based study in Germany to directly compare the treatment patterns and economic burden of ATX and LA-MPH monotherapy. These data were derived from patients covered by various types of insurance (statutory and private insurance, family member dependants and retirees), and from all geographic regions of Germany. Previous retrospective studies of ADHD treatment in Germany were conducted using claims data from a specific health insurance plan or geographic area [6, 12, 17].

Strict inclusion and exclusion criteria were used in the current study to ensure selection of only children/adolescents with a definite diagnosis of ADHD who had received ATX or LA-MPH monotherapy at index. As ATX and LA-MPH have different mechanisms of actions and may be used in different lines of therapy [1, 35, 36], substantial efforts were made to ensure that the two cohorts were as similar as possible in terms of available observed variables. Rigorous methodology (propensity score matching and multivariate modelling) was employed throughout to ensure comparability of cohorts and minimize bias, which is reflected in the large attrition observed between the initial sample selection and the matched treatment cohorts.

Another study strength was restriction of the treatment pattern analysis to include only novel initiators. This subset of patients had not received any ADHD-indicated medications (ATX, LA-MPH or SA-MPH) during the 12-month baseline period. The use of novel initiators alone to evaluate treatment outcomes avoids biases that are associated with data from ongoing treatment users (or so-called ‘prevalent users’) [37]. These biases result in difficulties in both identifying events that occur early in the course of therapy and controlling for potential confounding variables [37]. However, this methodology is likely to generate conservative estimates of treatment patterns as persistence among novel initiators is expected to be lower than for ongoing treatment users.

This study was designed to specifically focus on the burden of children/adolescents with ADHD who received ATX or LA-MPH who had been treated as outpatients, prescribed medications and/or referred for inpatient management. As such, we cannot evaluate the costs associated with multidisciplinary services such as psycho-education, psychotherapy, or complex case management. These non-pharmacological interventions are central to the maintenance of effective long-term therapy and contribute substantially to overall treatment costs [4, 12]. It is likely that many children/adolescents in this study would have received concurrent behavioural therapy [4, 38] but we are unable to predict whether the associated costs would differ between the treatment cohorts.

We also acknowledge several weaknesses inherent to our data source and methodology. The data used were obtained from physicians in general/internal medicine, paediatrics, psychiatry and neurology in Germany. We believe that the vast majority of patients with ADHD would have been managed by physicians in these specialties; however, it is possible that some patients may have received treatment from other practitioners [6, 7, 17]. We also assumed that the data provided by healthcare professionals were current and complete, although we fully acknowledge that this was an administrative database, as noted below. If incorrect diagnoses or pharmacy codes were listed in the medical records, or the record was incomplete, then our findings could be inaccurate.

The data were derived from an EMR database and were collected primarily as a clinical registry rather than as a retrospective outcomes research tool. As such, neither prescription fills nor medication consumption could be verified. However, rigorous inclusion criteria were used to ensure that only data on patients who were actively engaged in treatment were evaluated. Patients were required to have received at least two prescriptions for their index medication to have been included in our study. This methodology is likely to represent patients who consistently took their medications, and is likely to lead to conservative estimates of treatment patterns.

We acknowledge that the preferential inclusion of patients who received both ATX and LA-MPH during the selection window into the ATX-indexed group may have introduced a selection bias. Because of the methodology used to match treatment cohorts in this analysis, it is possible that the ATX-indexed cohort may be more representative of real-world practice, where patients have often been exposed to multiple products, than the MPH-indexed cohort. It must also be noted that the novel initiator patients may not be truly ‘medication-naïve’ as they could have received ADHD medication prior to the specified 12-month baseline period.

The discontinuation date was defined as the date immediately following a 30-day gap in the index medication (except for summer drug holidays). Therefore, patients may have discontinued their medication earlier than estimated. However, we consider that the impact of this potential underestimate is minimal as prescriptions in this specific German EMR database typically provided a 60-day supply.

The current study is also subject to common limitations of real-world data-based studies. Unobserved variables may confound the outcomes in retrospective cohort studies. In propensity score-matched studies, patients can be balanced only by known cohort characteristics. There are also limitations relating to the real-world data source, as the full range of clinical symptoms of ADHD was poorly recorded in this EMR database.

## Conclusions

This retrospective, propensity score-matched cohort study provides important real-world data on treatment patterns, HRU and cost among children and adolescents with ADHD who received ATX or LA-MPH in Germany. ATX-indexed novel initiators had longer persistence but higher switching rates than did LA-MPH-indexed novel initiators. The ATX-indexed cohort required more prescriptions and physician visits, and incurred higher total healthcare costs compared with the matched LA-MPH cohort. Thus, our data suggest that the use of ATX for the treatment of children/adolescents with ADHD in Germany may be associated with greater HRU and costs versus LA-MPH. Additional research is required to compare treatment patterns, resource use and the associated economic burden of ADHD among children/adolescents treated with ATX versus other stimulant medications in Europe, and the wider societal costs associated with ADHD.
